# Upcycling of Acid-Leaching Solutions from Li-Ion Battery Waste Treatment through the Facile Synthesis of Magnetorheological Fluid

**DOI:** 10.3390/molecules28062558

**Published:** 2023-03-11

**Authors:** Magdalena Abramowicz, Magdalena Osial, Weronika Urbańska, Mikołaj Walicki, Sławomir Wilczewski, Agnieszka Pregowska, Katarzyna Skórczewska, Piotr Jenczyk, Magdalena Warczak, Marcin Pisarek, Michael Giersig

**Affiliations:** 1 Faculty of Chemistry, University of Warsaw, Ludwika Pasteura 1 Street, 02-093 Warsaw, Poland; 2 Institute of Fundamental Technological Research, Polish Academy of Sciences, Pawinskiego 5B Street, 02-106 Warsaw, Poland; 3 Department of Environmental Protection Engineering, Faculty of Environmental Engineering, Wrocław University of Science and Technology, Wybrzeze Wyspianskiego 27, 50-370 Wroclaw, Poland; 4 Faculty of Physics, University of Warsaw, Ludwika Pasteura 5, 02-093 Warsaw, Poland; 5 Faculty of Chemical Technology and Engineering, Bydgoszcz University of Science and Technology, Seminaryjna 3, 85-326 Bydgoszcz, Polandmagdalena.warczak@pbs.edu.pl (M.W.); 6 Institute of Physical Chemistry, Polish Academy of Sciences, Kasprzaka 44/52, 01-224 Warsaw, Poland

**Keywords:** environment protection SPION, battery waste, toxic waste management, direct recycling, sustainability, circular economy, critical raw materials

## Abstract

The rapidly growing production and usage of lithium-ion batteries (LIBs) dramatically raises the number of harmful wastes. Consequently, the LIBs waste management processes, taking into account reliability, efficiency, and sustainability criteria, became a hot issue in the context of environmental protection as well as the scarcity of metal resources. In this paper, we propose for the first time a functional material—a magnetorheological fluid (MRF) from the LIBs-based liquid waste containing heavy metal ions. At first, the spent battery waste powder was treated with acid-leaching, where the post-treatment acid-leaching solution (ALS) contained heavy metal ions including cobalt. Then, ALS was used during wet co-precipitation to obtain cobalt-doped superparamagnetic iron oxide nanoparticles (SPIONs) and as an effect, the harmful liquid waste was purified from cobalt. The obtained nanoparticles were characterized with SEM, TEM, XPS, and magnetometry. Subsequently, superparamagnetic nanoparticles sized 15 nm average in diameter and magnetization saturation of about 91 emu g^−1^ doped with Co were used to prepare the MRF that increases the viscosity by about 300% in the presence of the 100 mT magnetic fields. We propose a facile and cost-effective way to utilize harmful ALS waste and use them in the preparation of superparamagnetic particles to be used in the magnetorheological fluid. This work describes for the first time the second life of the battery waste in the MRF and a facile way to remove the harmful ingredients from the solutions obtained after the acid leaching of LIBs as an effective end-of-life option for hydrometallurgical waste utilization.

## 1. Introduction

Global social and economic development determines both the number of products introduced to the market and the modern technologies in which critical raw materials play a crucial role, e.g., lithium and cobalt. These strategic metals are contained in lithium-ion batteries (LIBs), which dominate in electronic devices and electric vehicles due to their high power density and long life span. Consequently, battery waste disposal became a challenging issue while the number of customers rapidly grew. Thus, developing an entirely zero waste approach and recovery of raw materials is highly important [1,2]. Traditional LIBs utilization—namely taking, using, and disposing of them—is unsustainable. Therefore, the possibility of post-process waste management to improve sustainability is constantly being sought, thus closing the life cycle of raw materials following the principles of the circular economy (CE) model [3]. It is known that LIBs waste powder is a valuable source of various kinds of substances, in particular metals like Li, Co, Ni, Mn, and carbon. In the case of incorrect recovery processes, all these ingredients are highly harmful to the environment [4]. The landfilling and incineration of LIBs may provide soil and underground water contamination. Thus, the recycling process provides the reintroduction of the battery compounds into the economic cycle, and in this way, the demand for raw materials decreases [5]. However, the main emphasis on development is put down on the expansion of the material recovery technologies, and a relatively small effort is paid to analyze the influence of the environment and economic cost [6,7]. The essence of battery waste management is to provide efficient raw material recovery and extend the battery life cycle with minimal processing [8].

As the battery market dramatically increases due to the electrification of the transportation sector in the world, the effective recycling of LiBs at any cost is very important to protect the environment. The commonly used methods of direct recycling, which enable recoveries of valuable materials like Li, Co, and Ni, are pyrometallurgy and hydrometallurgy [9], where most pyrometallurgical methods are not able to recover Li [10]. Li is recovered as LiCO_3_ in China and South Korea and used for cathode compounds [11]. Compared to pyrometallurgy, hydrometallurgy has numerous advantages such as the ability to process different types of waste batteries simultaneously, lower process temperatures, the possibility of use on a small industrial scale, a significant reduction in the emission of potential pollutants, and the high efficiency of recovering valuable metals. These contribute to its wide applications in industry and laboratory studies [12]. Nevertheless, even after the selective recovery step, some Li and Co and all other metals remain in the solutions and require proper management, which is a tremendous technological challenge due to the solution’s properties (very low pH) and polymetallic composition. Thus, an efficient and environmentally friendly process of valuable materials recovery and separation is needed [13].

Furthermore, given the severe economic impact associated with the recirculation and recovery of materials from spent batteries and accumulators, the development of materials using them is highly important. The efficiency of LIBs leaching is strongly dependent on leaching agent composition, and the process temperature, time, and dosage [14,15,16,17,18] provide higher efficiency in the leaching process. Before leaching, one can apply the pretreatment of active electrode materials [19]. Considering the economic and ecological aspects (mainly the list of Critical Raw Materials [20] and the gradual introduction of legal regulations related to electromobility), there is a considerable need for recovering materials in the chemical leaching of waste lithium-ion batteries. A huge effort has been made to optimize the process of leaching to recover critical metals contained in spent LIBs, such as lithium and cobalt [17,21,22]. As a result of chemical leaching, solutions containing various metals are obtained. It is possible to separate them selectively and bring them to a solid form, but most often it focuses only on critical metals, such as the aforementioned lithium or cobalt [7,17,20]. Thus, even if some of the metals are recovered selectively for use in solid, commercial products, there remains a problem related to the development of the application of the remaining toxic, polymetallic solutions obtained after the leaching process. One of the promising proposals for the management of such liquids can be the production of magnetorheological liquids used in the automotive industry as in intelligent drive systems or damping systems described in this manuscript. In this way, liquid and harmful waste solutions after hydrometallurgical recovery of metals from spent Li-ion batteries can be reused in a closed loop of raw materials. Such an approach means that we propose a method of recycling the waste after the recycling of Li-batteries, which is of increasing importance due to the ever-growing usage of batteries in developed economies.

In view of problems associated with solid LIBs-based wastes and post-treatment acid-leaching wastes generated by portable electronics and the automotive sector, this paper presents the utilization of toxic solutions obtained from the acid-leaching of battery waste in innovative materials technology. Special attention is being paid to preparing a functional material through the zero-waste approach, where the harmful LIBs-based waste is fully recovered. In the first step, the battery waste was treated with an acid bath to recover valuable metals. Then, by-products—so-called acid leaching solutions (ALS)—were used as a source of valuable metals that can be incorporated into magnetic nanoparticles through co-precipitation, where the obtained material had different properties depending on the solution’s content during synthesis. Finally, the magnetic particles doped with metals from the battery waste acid leaching treatment were suspended in silicon oil, and the functional material—a Magnetorheological Fluid (MRF)—was obtained. To our knowledge, it is the first attempt to provide an effective way to recover heavy metal ions within the preparation of MRF. The proposed approach helps neutralize harmful acidic solutions containing cobalt within a facile wet co-precipitation, where superparamagnetic iron oxide-based nanoparticles are obtained. Consequently, an environment-friendly and sustainable battery waste leaching method has been developed.

## 2. Results and Discussion

### 2.1. UV-Vis Analysis of the Post-Synthesis Solution

**Figure 1:**
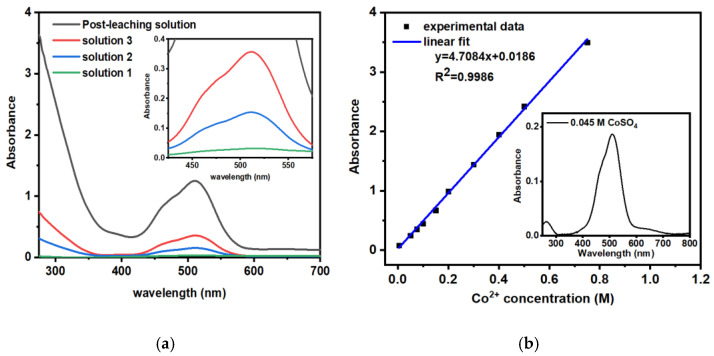
(**a**) UV-vis spectra of post-leaching solution and solutions 1–3 obtained after SPIONs synthesis (measurements without SPIONs in solution) and (**b**) calibration curve of Co^2+^ concentration, where inset presents the 0.045 M CoSO_4_ reference solution.

The post-treatment acidic solution contains several metal ions that can be harmful to health [15]. One of them is cobalt(II), which has good solubility in water and can cause a negative effect on the integrated control of environmental pollution. Therefore, there is a deep need for its management to effectively protect the aquatic environment. As the goal of this work was the incorporation of harmful heavy metal ions from ALS obtained after battery waste powder treatment, at first, the UV-vis studies were performed directly after the synthesis to determine the rate of the Co^2+^ removal from the ALS. The precursor bath had a pink color, indicating the presence of pink color Co^2+^ ions, while after the precipitation, the bath was decolorized, and the dark precipitate formed. As the composition of the post-leaching bath was estimated within ICP-OES, the UV-vis was used complementarily to determine if the chromophores were incorporated into the structure of the deposited compounds. The black curve corresponds to the precursor solution that was used in the synthesis of nanoparticles—a source of valuable metals (Figure 1a). Based on the literature, it can be concluded that the bands correspond to the metal ions like cobalt [23,24,25], manganese [26], or other metal ions. The following spectra were measured for the solutions left after nanoparticle extraction. Figure 1a shows the spectra recorded for the fresh ALS after the treatment of LIBs and the filtration from the solid residues, and the spectra for the solution collected after the co-precipitation of iron oxide-based magnetic particles. As can be seen, a broad band in the region of 550–450 nm is recorded, and its intensity lowers for samples 1–3. To determine the content of Co^2+^ ions in the bath, the CoSO_4_ reference solution was prepared with different concentrations. Figure 1b shows the calibration curve for a such solution. The inset reveals the spectrum for 0.045 M solution, revealing a shape similar to ALS. Based on the calibration curve, the content of Co^2+^ in the post-leaching solution was determined to be about 0.268 M and decreased to 0.073 M, 0.025 M, and 0.003 M for samples 1–3 (made from solutions 1–3), respectively.

The more post-leaching waste was incorporated into nanoparticles, the less was left in the solution, hence the absorbance decreases. It can be concluded that ingredients of ALS can be easily removed from the solution with their incorporation into magnetic nanoparticles. The increase in the post-leaching solution content in the synthesis bath leads to the partial incorporation of waste ingredients into the nanoparticles.

### 2.2. Synthesis of Magnetorheological Fluid

Based on the UV-vis measurements, the procedure ascribed for sample 1 (obtained from solution 1) was used to prepare MRF. It is due to the highest purity of the post-synthesis solution, so the highest removal of harmful chemicals from the ALS. Based on the procedure described above, 30 g of dry nanoparticles were prepared. The nanoparticles were collected on the magnet and the water was removed. Then, 50 mL of silicon oil was added, and the suspension was mixed mechanically at 400 rpm at 120 °C on the hot plate for 2 h to remove the moisture. When the water evaporated, the temperature on the hot plate was decreased to 60 °C and the stirring was turned off. Here, the silicon oil was added up to 100 mL of the suspension and the suspension was stirred again. Then, the 3 mL of oleylamine was carefully added dropwise and the suspension was stirred at 1000 rpm for 1 h, and left for stirring at 400 mg overnight at the remaining temperature.

### 2.3. Morphology Studies

**Figure 2:**
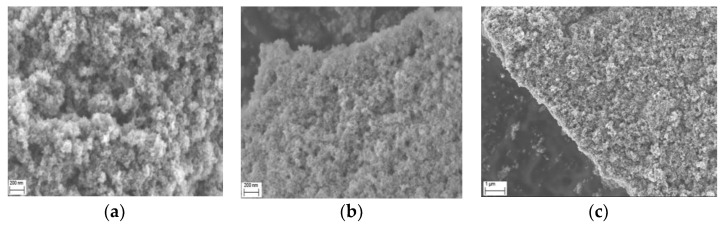
SEM images of (**a**) sample 1 with 1 mL waste ALS, (**b**) sample 2 with 2.5 mL waste ALS, and (**c**) sample 3 with 5 mL waste ALS.

**Figure 3:**
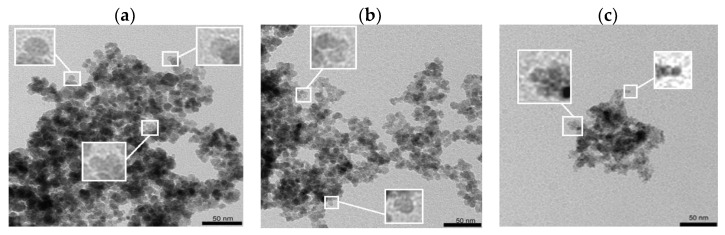
TEM images of (**a**) sample 1 with 1 mL post-leaching solution, (**b**) sample 2 with 2.5 mL post-leaching solution, and (**c**) sample 3 with 5 mL post-leaching solution.

The morphology of particles doped with the ions obtained within the acid leaching of the waste battery powder was investigated with SEM and TEM. As can be seen in Figure 2 presenting SEM images, depending on the dopant content, the morphology of particles changes. The size of the particular grain for sample 1 (Figure 2a) is much lower than for samples 2 (Figure 2b) and 3 (Figure 2c), which contain more dopants. The product forms agglomerates that are caused by the drying of the sample after the synthesis and washing (the wet water-based suspension was placed onto the copper conductive tape and dried on air overnight).

As the SEM images could only deliver a general overview of the morphology of the prepared samples, the following studies were focused on the precise determination of the shape and size of obtained particles within the TEM. When the post-leaching solution consisted of 5% in volume for sample 1 (19 mL of iron salts over 1 mL of post-leaching solution), the obtained nanoparticles had a spherical shape and size up to 12 nm average, see Figure 3a. Their size and shape are similar to the data obtained in the literature for bare iron oxide nanoparticles [27,28]. Sample 2 presented in Figure 3b has a similar shape and size, while a slightly smaller structure can be distinguished. The increase in the post-leaching solution added to the bath for the synthesis of nanoparticles leads to a drop in the size of the product. As can be seen in Figure 3c, nanoparticles have a size even twice smaller than for samples 1 and 2. This can be caused by the different ionic strength during synthesis and the lower pH at the initial point—before the co-precipitation.

Depending on the composition of the liquid battery waste, the synthesis way of nanoparticles, and experimental conditions during synthesis, the morphology of particles obtained from hydrometallurgical waste can differ. For example, Dun et al. obtained Co_1+*x*_Fe_2−2*x*/3_O_4_, where x ranged from 0 to 0.2 using the sol–gel auto-combustion method, where the morphology studies show the fractured inner surface of the sintered cylindrical rods in a few microns in diameter [29]. In work presented by Moura, CoFe_2_O_4_ nanostructures are sized from 10 to 50 nm [30], while Co_0.8_Fe_2.2_O_4_ prepared in another work from LIBs cathode leached with acids have diameters of ~50–120 nm [31,32]. Here, the CoFe_2_O_4_ and Co_0.87_Ni_0.13_Fe_2_O_4_ obtained by co-precipitation from the hydrometallurgical waste are also spherical, but the nanoparticles have the size of about 100 nm [32,33], demonstrating the NiCo ferrite magnetic nanoparticles made with wet synthesis from the solutions obtained from acid leaching of spent lithium-ion batteries and nickel-metal hydride batteries, where the obtained structures have a diameter ranging from 29 to 60 nm [34]. Next, Mn_0.6_Zn_0.4_Fe_2_O_4_ co-precipitated from Mn-Zn post-leaching battery waste form clusters from tens to hundreds of microns [34,35], showing Mn-Zn ferrite structures sized up to 20 nm [34]—similar to the structures synthesized in presence of bacteria [36]. The granular shape of obtained samples in this work is in good agreement with the discussed literature, while their size of below 15 nm on average is similar to the CoFe_2_O_4_ spherical nanoparticles sized about 20 nm obtained with sol–gel, co-precipitation, and microwave-assisted hydrothermal methods [37].

### 2.4. Magnetic Properties 

**Figure 4:**
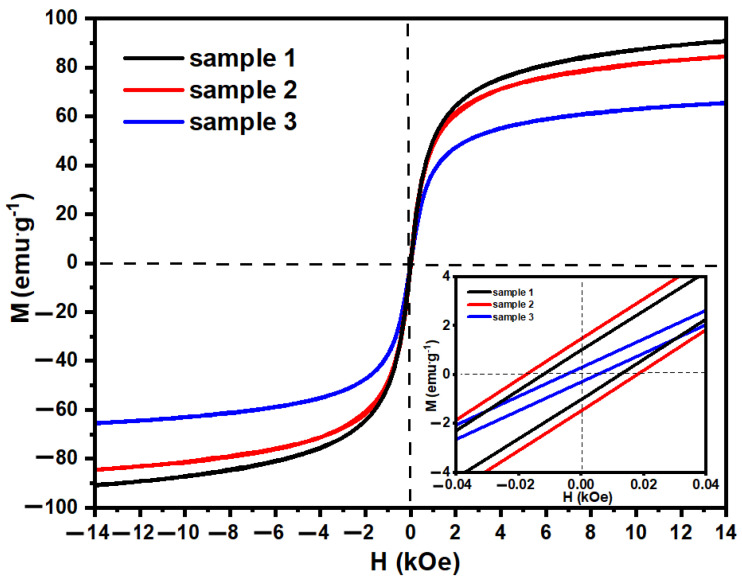
Magnetic change in function of magnetic field for samples 1–3.

As the nanoparticles are planned to be used in the MRF, their magnetic properties were investigated with magnetometry. As can be seen in Figure 4, the saturation magnetization differs for the particles obtained in different conditions, where the highest values are observed for sample 1. Here, the saturation magnetization (M_s_) is about 91 emu g^−1,^ and the coercivity (H_c_) is below 20 Oe. The M_s_ values for samples 1 and 3 are 84.4 and 65.6 emu g^−1^, respectively. The values of the saturation magnetization and the shape of the hysteresis for sample 1 are similar to the characteristics of the bare iron oxide nanoparticles, where M_s_ is about ~90 emu g^−1^ [38,39,40,41]. According to the values for the nanoparticles obtained from the hydrometallurgical waste, Sankaran et al. present ferromagnetic Co_0.87_Ni_0.13_Fe_2_O_4_ and CoFe_2_O_4_ nanoparticles with M_s_ of about 62 emu g^−1^ and a coercivity of about 1420 and 760 Oe, respectively [32]. Dun et al. demonstrate different values of M_s_ and H_c_ for CoFe_2_O_4_ nanoparticles from LIBs waste obtained with different synthesis methods ranging M_s_ from 71.2 to 77.3 91 emu g^−1^ and H_c_ from 26.4 to 272.7 Oe [29]. The inset presented in Figure 4 shows the narrow hysteresis loop suggesting the superparamagnetic character of the nanoparticles. The results presented here are in good agreement with the literature, showing the potential of synthesized structures to be used in various fields, where the superparamagnetism of nanoparticles is required. Despite the decrease in the magnetization of the particles with an increase in the dopant, the samples still reveal superparamangetism, which makes them attractive for application in many sectors, including magnetorheological fluids.

### 2.5. Chemical Composition Studies

**Figure 5:**
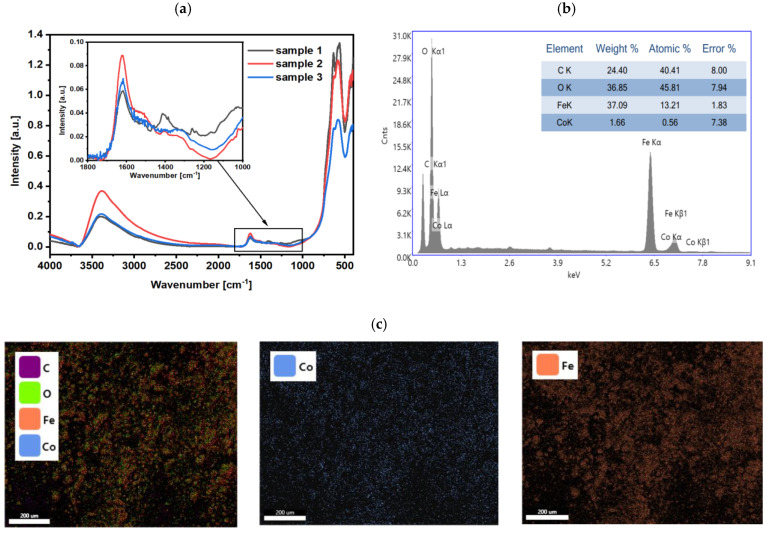
(**a**) FT-IR spectra of samples 1–3, (**b**) EDS spectrum for sample 1, and (**c**) distribution of C, O, Fe, and Co in sample 1.

In the next step, the characterization of the chemical composition of the nanoparticles within the FT-IR for samples 1–3 in the wavenumber from 400 to 3600 cm^−1^ was made, see Figure 5. The range below 700 cm^−1^ is characteristic of the vibrations in the metal oxide structures. The range 900–1700 cm^−1^ is characteristic of the presence of organic compounds in the materials, while the range 2800–3600 cm^−1^ is useful to determine the presence of –OH and –CH groups in the measured materials.

As can be seen in recorded spectra, depending on the amount of the waste ALS for the iron oxide doping, the composition of the final product varies. It can be noticed that absorption increases when the amount of ALS increases. Three characteristic peaks can be observed in the range of the lowest wavenumbers. The peaks at 564 cm^−1^ and approx. 625 cm^−1^ can be identified as the Fe-O vibrations from Fe_3_O_4_ [42,43]. For cobalt(II, III) oxide, the Co-O bond peak should be assigned at 570 cm^−1^, which is probably because the peak at 564 cm^−1^ is not sharp and extends over 570 cm^−1^ [44]. It can be concluded that the admixture metals have been successfully incorporated into the structure of the SPIONs. In this range, the spectra of syntheses are not similar. This can be related to the taken sample and the amount of admixture. It was possible to assign bonds that are characteristic of approx. 1120 cm^−1^ C-O vibration [45], 1400 cm^−1^ vibration, which is in correlation to the C-O bond in a carboxylic acid. The vibration at approx. 1620 cm^−1^ can be attributed to the C = O bond [42]. The identified vibrations allow the determination of carboxylic acids bounded with nanoparticles. Two characteristic peaks can be observed in the left-most range. Peaks that can be observed at 3150 cm^−1^ and 3350 cm^−1^ can be attributed to the O-H groups which correspond to the presence of carboxylic acid [42,45]. Obtained results confirm the successful incorporation of the organic compounds from the leaching bath into the nanoparticles.

The presence of organic compounds in the obtained nanostructures was also confirmed with the zeta potential studies. As the bare reference iron-oxide particles without the ALS dopants have very low surface potential, close to 0 mV, the results for ALS-doped samples are different. The surface potential for samples 1–3 is as follows: −19.2 ± 2.1 mV, −26.4 ± 2.7 mV, and −29.9 ± 3.4 mV. It confirms the presence of negative functional groups on the surface of nanoparticles, complementarily to FT-IR results.

Based on the FT-IR, magnetometry, and zeta potential results, the sample—having the highest value of M_s_—was also studied with X-ray dispersive spectroscopy (EDS) to determine the distribution of the elements in the nanoparticles. As can be seen in Figure 5b, the peaks characteristic to the Fe, O, C, and Co can be seen. According to the carbon content in the sample, it cannot be determined precisely for the traces of carbon in the tape that was used to immobilize the sample onto the alumina holder. As expected from the synthesis, the sample contains Fe, O, and Co, where the Co consists of about 0.56% atomically, which is uniformly distributed in the sample.

Complementarily to the FT-IR and EDS analysis, X-ray Photoelectron Spectroscopy (XPS) was used to determine the chemical composition of the obtained nanoparticles. In addition to the main components—iron oxides—XPS spectra revealed the presence of cobalt and manganese in the particles, confirming the effective recovery of these metals from the acid—leaching bath used to treat the battery waste. Besides these metals, carbon is also found to confirm the modification of the surface with organic compounds, particularly the glutaric acid that was used as a reducing agent in the acid-leaching procedure of battery waste. As the saturation magnetization is the highest for sample 1, it was chosen for XPS to check the content of leached metals and/or possible organics that were used as mild leaching agents.

**Figure 6:**
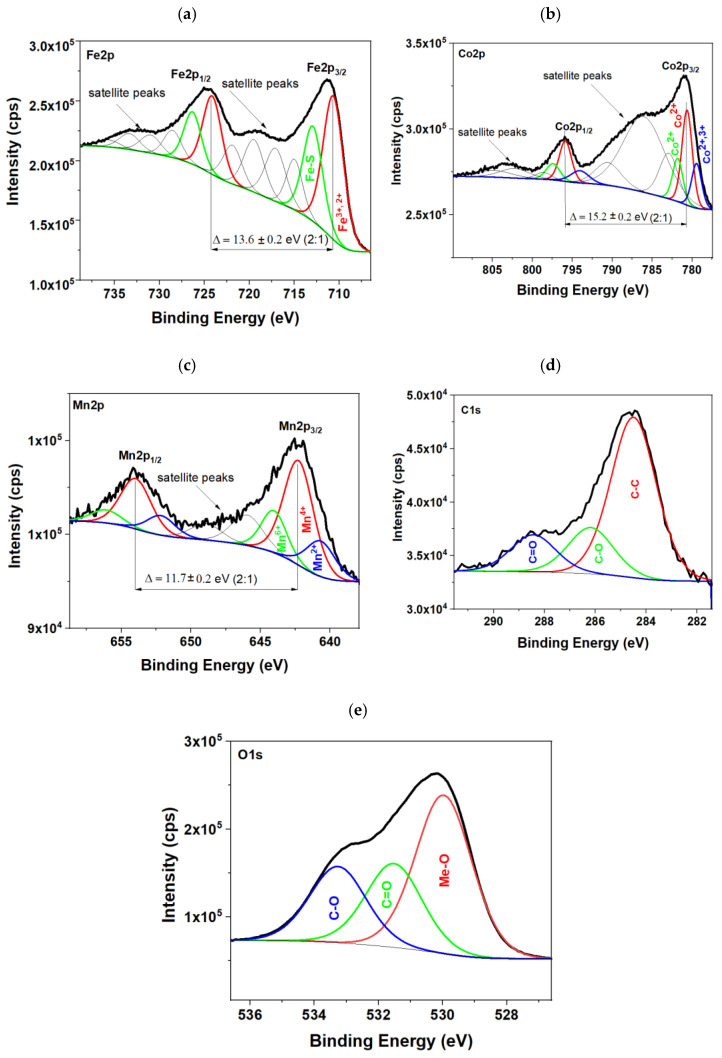
XPS spectra for sample 1 chosen for MRF synthesis, in particular, (**a**) Fe 2p, (**b**) Co 2p, (**c**) Mn 2p, (**d**) C 1s, and (**e**) O 1s core level spectra.

According to the iron-based compounds present in the sample, Figure 6a shows Fe 2p core-level spectra. Two characteristic signals can be distinguished in the spectrum, which comes from spin-orbitals 3/2 and 1/2. Deconvolution of this peak showed that the first pair of peaks at about 710.7 eV and 724.1 eV corresponds to Fe-O bonding in Fe_3_O_4_ mainly as an Fe^3+^ ion [46,47]. The second pair at about 712.9 eV and 726.3 eV can be ascribed to the Fe-S bonding from the traces of post-leaching residues in sulphuric acid media [48,49]. There are also clear satellite lines in the spectrum, which clearly indicate that Fe is in the oxidized form [48]. The Co 2p core-level spectrum shown in Figure 6b reveals many peaks, indicating a complex structure of the obtained nanoparticles. As in the case of the Fe 2p peak, the Co 2p signal indicates the appearance of Co-O bonds in the investigated material. If we look at the binding energy range for Co 2p_3/2_, we can distinguish peaks that relate to Co_3_O_4_ (779.4 eV), CoO (780.6 eV), and Co(OH)_2_ (781.8 eV) [47]. Similar results were presented by Dun et al. for the cobalt ferrites obtained from LIBs [37]. The presence of Co^2+^ in individual oxide forms of this element also confirms the location of the Co 2p_3/2_ and Co 2p_1/2_ satellite lines, even though the shape of the Co 2p spectrum is unusual [50]. This is related to the interaction of the X-ray beam with the sample matter [51]. Figure 6c shows the Mn 2p core level spectrum at which the peaks at 640.7 eV, 642.3 eV, and 644.0 eV correspond to the Mn 2p_3/2_ state [46]. Next, the group of peaks at 652.2 eV, 654.0 eV, and 656.1 eV can be ascribed to Mn 2p_1/2_ [52]. The location of the detected Mn 2p_3/2_ signals relative to the binding energy suggests the presence of manganese oxides: MnO_2_, MnO, and manganese ferrate [47,53]. The C 1s spectrum in Figure 6d was a curve fitted into three different peaks through XPS measurements. The binding energies at about 284.5 eV, 286.2 eV, and 288.4 eV can be assigned to the C–C, C–O, and C = O groups from acidic groups [48,54], respectively. The O 1s spectrum seen in Figure 6e shows peaks at 530.0 eV, 531.5 eV, and 533.3 eV, corresponding to metal oxides and C = O and C-O bonding, respectively [46,48].

The XPS revealed the complex composition of obtained nanoparticles. Besides the metal oxides, the nanoparticles contain carbon-based compounds on the surface. Based on the obtained results it is clearly seen that the nanoparticles are successfully doped with the ingredients from the leaching bath.

### 2.6. TGA Analysis of the MRF

**Figure 7:**
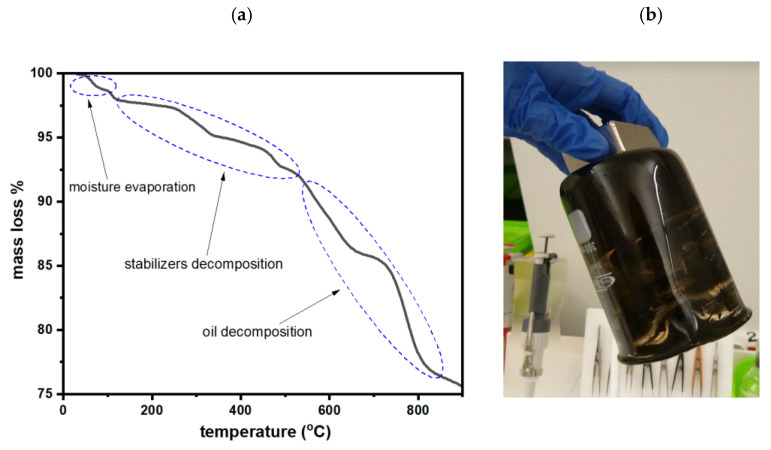
(**a**) Thermogravimetric curve of the MRF, and (**b**) the MRF attracted by the magnet and collected at the bottom of the beaker.

Having confirmed the successful synthesis of the nanoparticles, the MRF obtained with the use of sample 1 was investigated with thermogravimetry, where the content of organic ingredients was determined. As can be seen in Figure 7a, mass loss is observed along with the increase in the temperature. The initial drop in the curve up to 100 °C corresponds to the water evaporation from the sample. The following decrease in the mass can be ascribed to the decomposition of the organic stabilizer, where the organic coat consists of approx. 10%. Then, the subsequent increase in the temperature to above 300 °C can be ascribed to the decomposition of the oil as well as to the organic compounds from the nanoparticles being decomposed.

### 2.7. Viscosity Measurements

**Figure 8:**
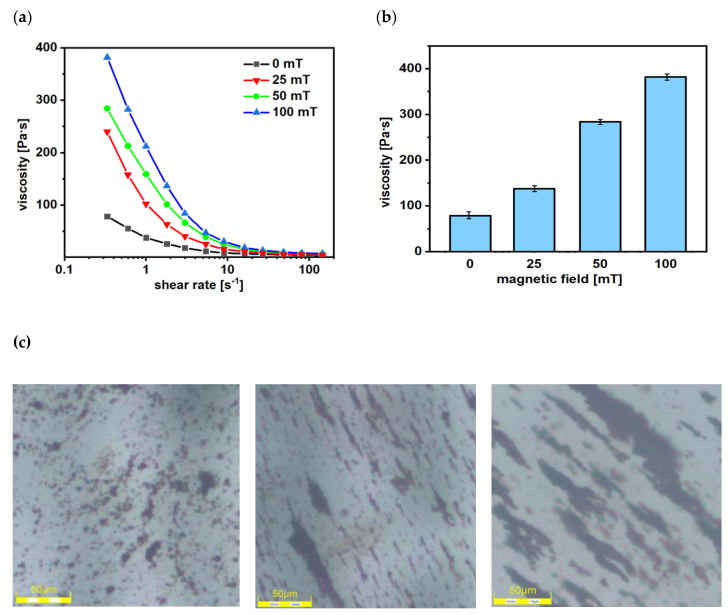
The change in the MRF’s viscosity in the function of (**a**) shear rate, (**b**) amplitude of magnetic field at a shear rate 0.3 s^−1^ (0, 25, 50 provided by coils, and 100 mT provided by magnets), and (**c**) optical microscope images of the MRF without magnetic field (**left**), with 25 mT fields (**middle**), and 50 mT (**right**).

In order to define the rheological properties of prepared MRF the viscosity change under the magnetic field was investigated. The MRFs were examined using a rotating rheometer (Rheotest 2.1, Germany) that was dedicated to performing experiments under the external magnetic field. As can be seen in Figure 8a, the MRF is thickening with the increase in the magnetic field. The shear thinning is observed, and almost all MRFs significantly increase their viscosity upon the application of a magnetic field. The effect of the field is most profoundly observed in the low shear rate regime showing up to a 300% rise and then becoming almost insignificant as the shear rate is increased. The size of this effect is similar to SPIONs made of pure iron oxide [38]. Figure 8b presents the change in the viscosity of MRF over the amplitude of the magnetic field, where the shear rate was about 0.3 s^−1^. The obtained MRF is able to change its rheological and micromechanical properties under the application of the external magnetic field [55]. Increased viscosity in presence of the magnetic field is an effect of the chain-like structure formation in the MRF. Figure 8c shows the increase in the length and width of these chains with the increased magnetic field.

Recycling polymetallic spent batteries plays a crucial role in waste management and the sustainable use of raw materials. Nevertheless, the applied industrial technologies based on pyrometallurgy and hydrometallurgy are not waste-free. Despite recovering critical raw materials such as lithium and cobalt, the process still leaves various types of waste that require proper management. The recycling of spent Li-ion batteries is based on the acid leaching of the electrode powder in order to leach out the metals contained therein; however, this process generates tremendous amounts of harmful waste that need to be managed. One of the solutions is an application of the acid waste into the magnetic nanoparticles, which offers a wide range of applications.

## 3. Materials and Methods

We briefly present the production process of the MRF, which is based on compounds prepared in [56]. For the MRF preparation, solutions after the acid leaching of polymetallic electrode powder were obtained from the mechanical treatment of spent lithium-ion batteries from laptops of various brands in the presence of a leaching agent. Then, after the spent battery powder treatment, the post-leaching solution was used as a source of metals to be incorporated into the structure of the superparamagnetic iron oxide nanoparticles (SPIONs).

The leaching procedure was carried out by using Nitric acid HNO_3_ with 65% for the pre-treatment of spent battery waste supplied from POCH, Gliwice, Poland. Sulfuric acid H_2_SO_4_ with 96% analytical grade and hydrogen peroxide H_2_O_2_ with 30% analytical grade were ordered from STANLAB, Lublin, Poland. Sulfuric agent was used for leaching and hydrogen peroxide was used as the reducing agent. Water used for synthesis and washing was purified with a Milli-Q Merck filtering system, where water had a resistance of about 18.2 MOhm^.^cm at 25 °C. In turn, Iron (III) chloride hexahydrate FeCl_3_·6H_2_O (analytical grade) was purchased from WARCHEM, Warsaw, Poland, and iron (II) chloride tetrahydrate FeCl_2_·4H_2_O was supplied from ACROS Chemicals. These salts were used for the synthesis of iron oxide-based nanoparticles. The 25% ammonia solution NH_4_OH that was applied to precipitate nanoparticles from the iron salt-based solution was supplied from POCH, Gliwice, Poland. Silicon oil for high temperatures was purchased from ACROS Organics. Adipoyl chloride 98% grade was supplied from Sigma-Aldrich.

The metal concentrations in the solution were determined using Inductively Coupled Plasma Optical Emission Spectrometry (ICP-OES, Agilent 720). The morphology of SPION obtained within the co-precipitation method was examined by Scanning Electron Microscope (SEM, Hitachi S-4800-Japan) and Transmission Electron Microscope (TEM, Jem 1400 Flash, Jeol-Japan). The elemental composition of the samples was determined by SEM-EDX (SM-6510LV, Jeol-Japan, X-Act, Oxford Instrument-England). The saturation magnetization of SPIONs was measured by a vibrating sample magnetometer (VSM) under the maximum applied field of 20 kOe at room temperature. Fourier Transform Infrared (FTIR) spectra of samples were recorded by Nicolet iS10, Thermo Scientific. The specially constructed test for viscosity changes in presence of a magnetic field was based on the rotary rheometer Rheatest 2.1 (Germany). The Rheometer was calibrated and adjusted with 30 Pa·s silicone viscosity standards (AMETEK Brookfield, Germany), obtaining 0.1 Pa·s measurement uncertainty. For the magnetorheological tests, the axial magnetic field was provided by either a current-controlled coil around the sample cylinder, generating fields up to 50 mT, or specially designed ring-shaped neodymium magnets (100 mT). Magnetic field strength was measured with a teslameter equipped with a Hall-effect probe (Lakeshore 475, USA). XPS measurements were performed using a Microlab 350 (Thermo Electron) with non-monochromatic Al K*α* radiation (*h*ν = 1486.6 eV) from an X-ray source.

### 3.1. Acid Leaching of Spent Battery Powder

Initially, the stream of spent Li-ion batteries was discharged and manually dismantled within the procedure described in [17,57,58]. Then, the battery-based powder was ground and separated mechanically from the plastic, metal, and paper solid pieces. In order to perform an initial quantitative and qualitative analysis of the powder material, 1 g of spent battery powder was mineralized with 10.0 mL 65% HNO_3_ for 5 h at 120 °C. As a result, metals were released from the solid material into the forming acidic solution. The content of particular metal ions was determined using Inductively Coupled Plasma-Optical Emission Spectrometer (ICP-OES).

**Figure 9:**
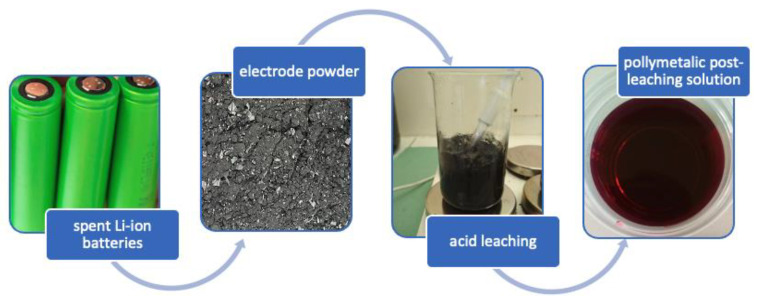
Schematic diagram of the solution prepared for use as a dopant.

The sample of electrode powder was leached in 1.5 M sulfuric acid (96%) + glutaric acid C_5_H_8_O_4_ (5% *w/v*) for 2.5 h at 90 °C. The suspension was also magnetically stirred (500 rpm), similar to the procedure described in [23], see Figure 9.

**Figure 10:**
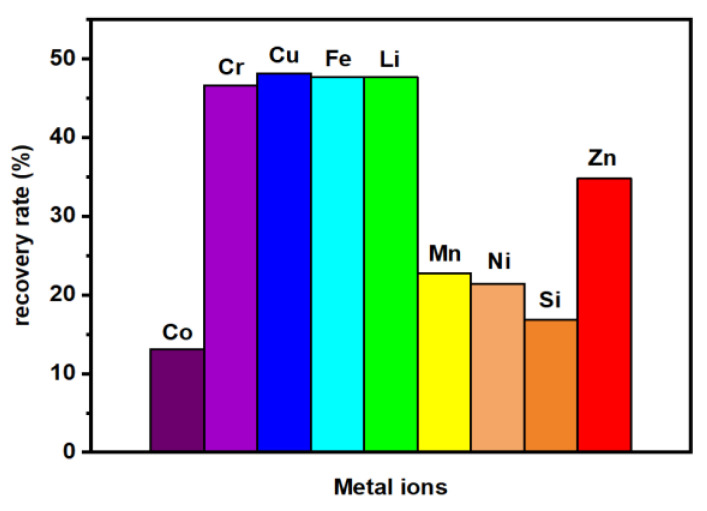
ICP-OES results for the recovery rate of particular ions after the acid leaching of spent battery waste. Note that the values are relative to the solution after acid leaching of the waste battery powder.

As a result, the valuable metals were released from the powder into the acidic solution. Then, the post-leaching solution was investigated within the ICP-OES technique to determine the recovery rate of metals—Co, Cr, Cu, Fe, Li, Mn, Ni, Si, and Zn, see Figure 10. As a result of leaching, a significant part of all tested metals was recovered, which is described in detail in the papers [56,59]. In this series of research, the highest recovery rates were received for seven of all the elements tested—Co, Cr, Cu, Mn, Ni, and Zn. Nevertheless, each of the obtained solutions is polymetallic, and the contained metals are in the form of ions that can be successfully incorporated into the structure of nanoparticles. Therefore, the solutions obtained after acid leaching of the electrode powder from spent Li-ion batteries were used for the co-precipitation of nanoparticles that can be used for the synthesis of the magnetorheological fluid.

### 3.2. The Application of the Spent Post-Leaching Solution to Prepare SPIONs

Initially, the iron oxide-based nanoparticles were synthesized for characterization under the following procedure using a wet co-precipitation method. First, 540 mg of FeCl_3_·6H_2_O were placed into the beaker and dissolved with 15 mL of water. Then, the beaker was left on the magnetic stirrer at 600 rpm to dissolve the iron (III) salt. Then, 190 mg of FeCl_2_·4H_2_O was added to the beaker and mixed for a few minutes until the salt was fully dissolved. Next, the post-leaching solution was added as follows: 1 mL (solution 1), 2.5 mL (solution 2), and 5 mL (solution 3), and then the water was added up to 20 mL to each synthesis bath. It is worth mentioning that the pH of the bath before the addition of the dopant was about 3, while for the particular dopants, it dropped from 3 to 2.5, 1.6, and 1, respectively, for the acidic pH in the ALS. Depending on the ALS content in the co-precipitation bath, the obtained samples were named as follows: sample 1 (from solution 1), sample 2 (from solution 2), and sample 3 (from solution 3). The beaker with the magnetic stirrer was set to heat at 70 °C for 15 min. Afterward, 25% NH_3(aq)_ solution was added to the mixture as a precipitation agent until the pH was 10.5 and was adjusted with several minutes of continuous mixing of the suspension. SPIONs were carried out under the given conditions for another 15 min. After that, the product was collected at the bottom of the beaker with a magnet and washed with Milli-Q water several times until obtaining a neutral pH. The mass of the final product was about 230 mg ± 5 mg.

## 4. Conclusions

In this work, we proposed for the first time magnetorheological fluids made from harmful liquid waste from LIBs treatment as the second life of toxic liquid battery waste containing heavy metal ions and organic compounds. The ALS obtained from the acid leaching containing heavy metal ions and organic and inorganic acids were neutralized and purified from both heavy metal ions and organic compounds through the synthesis of iron oxide-based magnetic nanoparticles. Through the use of ALS, the iron oxide nanoparticles doped with cobalt and glutaric acid were prepared using the co-precipitation technique, where the dopant content was controlled with the change in ALS volume in the synthesis bath. UV-vis spectra confirmed the decrease in Co^2+^ content in the ALS bath used for synthesis after co-precipitation from 0.268 M to 0.003 M, proving the removal of harmful ingredients from the hydrometallurgical waste. SEM and TEM studies revealed nanosized spherical structures, the size of which was dependent on the content of ALS in the synthesis bath. The increase in the volume of ALS reduces the size of obtained nanoparticles from 15 nm on average to even a few nm, and also decreased the magnetization saturation. Based on the morphology and magnetization results, the nanoparticles size was about 15 nm ± 4 nm, and the M_s_ of about 91 emu g^−1^ with a narrow hysteresis loop was chosen to be suspended in the viscous media as a magnetic carrier in the MRF. Obtained nanoparticles can be easily coated with organic agents to disperse them in viscous media like silicon oil. Therefore, it has been proposed to use these types of solutions resulting from the acid leaching of spent batteries to produce functional magnetorheological fluids that can be used in many fields, including dynamic energy dissipation, e.g., shock absorbers, electromagnetic clutch, etc.

## Data Availability

The datasets used and/or analyzed during the current study are available from the corresponding author at a reasonable request.

## Sample Availability

Samples of the compounds are available from the authors.
